# Concomitant Temperature Stress and Immune Activation may Increase Mortality Despite Efficient Clearance of an Intracellular Bacterial Infection in Atlantic Cod

**DOI:** 10.3389/fmicb.2018.02963

**Published:** 2018-12-04

**Authors:** Anett K. Larsen, Ingebjørg H. Nymo, Karen K. Sørensen, Marit Seppola, Rolf Rødven, María Pilar Jiménez de Bagüés, Sascha Al Dahouk, Jacques Godfroid

**Affiliations:** ^1^Arctic Infection Biology, Department of Arctic and Marine Biology, UiT – The Arctic University of Norway, Tromsø, Norway; ^2^Department of Medical Biology, UiT – The Arctic University of Norway, Tromsø, Norway; ^3^Department of Research and Development, UiT – The Arctic University of Norway, Tromsø, Norway; ^4^Unidad de Tecnología en Producción y Sanidad Animal, Centro de Investigación y Tecnología Agroalimentaria, Instituto Agroalimentario de Aragón, Universidad de Zaragoza, Zaragoza, Spain; ^5^German Federal Institute for Risk Assessment, Berlin, Germany

**Keywords:** *Brucella*, climate change, disease resistance, *Gadus morhua*, immunology, opportunistic infection, stress, temperature

## Abstract

The environmental temperature has profound effects on biological systems of marine aquatic organisms and plays a critical role in species distribution and abundance. Particularly during the warmer seasons, variations in habitat temperature may introduce episodes of stressful temperatures which the organisms must adapt to and compensate for to maintain physiological homeostasis. The marine environment is changing and predicted raises in water temperatures will affect numerous marine species. Translocation of pathogens follow migration of species and alternations in physical environmental parameters may have influence upon the virulence of pathogens, as well as the hosts immune responses. While pathogenicity of many true pathogens is expected to increase following climate induced temperature stress, the impact from environmental stressors on the occurrence and severity of opportunistic infections is unknown. Here we describe how thermal stress in the cold-water species Atlantic cod influenced the fish immune responses against an opportunistic intracellular bacterium. Following experimental infection with *Brucella pinnipedialis* at normal water temperature (6°C) and sub-optimal temperature (15°C), cod cleared the intracellular bacteria more rapidly at the highest temperature. The overall immune response was faster and of higher amplitude at 15°C, however, a significant number of cod died at this temperature despite efficient clearance of infection. An increased growth rate not affected by infection was observed at 15°C, confirming multiple energy demanding processes taking place. Serum chemistry suggested that general homeostasis was influenced by both infection and increased water temperature, highlighting the cumulative stress responses (allostatic load) generated by simultaneous stressors. Our results suggest a trade-off between resistance and tolerance to survive infection at sub-optimal temperatures and raise questions concerning the impact of increased water temperatures on the energetic costs of immune system activation in aquatic ectotherms.

## Introduction

The entire physiology of ectotherm vertebrates is influenced by their environmental temperature and changes in ambient temperature affect biological systems at all levels of organization, from molecule to organism (McCormick, 2011). Many species experience a wide range in habitat temperature due to seasonal variations and temperature plays a critical role in determining their distribution and abundance. Marine ecosystems are challenged as raised atmospheric carbon dioxide drives heating and acidification of the ocean (IPCC, 2014). Because of the increased ocean temperature and the lowered ocean pH, many marine species will find themselves in unsuitable environments, which challenge their survival (Bijma et al., 2013).

The Atlantic cod (*Gadus morhua*) has a pan-Atlantic distribution and, depending on season and location, experience a wide range of habitat temperatures from −1 to 19°C (Righton et al., 2010). Future environmental stress poses an increased risk for the Atlantic cod which is a species with low climate resilience, meaning that it has a low tolerance to temperature change (Lannig et al., 2004) and a high vulnerability to threats like over-fishing (Cheung et al., 2005). Depressed populations, as reported for several coastal cod stocks (MCS, 2015), are more likely to be affected by environmental change than thriving populations due to a reduced reproductive output and hence limited capacity for genetic and/or phenotypic variation (Sumaila et al., 2011).

The critical temperature for circulatory performance and stress responses in Atlantic cod is 16°C (Lannig et al., 2004; Pérez-Casanova et al., 2008a,b). This means that cod populations in the southernmost distribution area already experience stressful temperatures during the warm season of the year. The natural habitat of the coastal stocks of Atlantic cod are located at water depths of 0–400 m (ICES, 2006), and although they have the opportunity to migrate toward deeper and colder water in the warm seasons, some shoals of cod may have to withstand critical temperatures. Fry and young cod live in shallow waters (0–20 m) and rarely relocate to deeper areas before they are 2 years of age (IMR, 2017). In most areas inhabited by cod, mean ocean temperature is predicted to increase with 2–4°C by the year 2100 (IPCC, 2014). Such temperature rises may lead to a decrease, or in the worst case collapse, of several of the coastal cod stocks (Drinkwater, 2005).

Changes in water temperatures affect the fish immune system. Adverse effects arise at both high and low temperatures, with certain parameters being suppressed at the species lower temperature limits and others at upper temperature limits. Specific immune responses, in particular the production of antibodies and maturation of immunocompetent cells, are stronger and more efficient as temperatures increase above lower temperature limits (species specific immunologically “non-permissive” temperatures) (Le Morvan et al., 1998). Innate immune responses such as leukocyte respiratory burst activity and phagocytosis showed a reduction with increasing temperature in carp (*Cyprinus carpio*), while respiratory burst activity increased in rainbow trout (*Oncorhynchus mykiss*) and Atlantic cod (Nikoskelainen et al., 2006; Bowden, 2008). Humoral parameters found to be temperature-dependent in Atlantic cod are serum hemolytic activity, anti-protease activity, and natural antibody activity, of which the two first components are inhibited by higher temperatures, while the latter increases with elevated seawater temperatures (Magnadóttir et al., 1999). High water temperature has been shown to increase the transcription of immune relevant genes in Atlantic cod 24 h after a challenge, but with striking differences between bacterial and viral antigens (Hori et al., 2013).

At present, it is uncertain how temperature-induced changes in immune parameters will affect and/or modify the combined immune response toward infections, but latent bacterial, fungal and parasitic infections have been shown to cause disease in Atlantic cod kept at temperatures close to its upper thermal limit (Magnadóttir et al., 1999). In aquaculture, increased water temperature is a key stress factor in many disease outbreaks (Green and Haukenes, 2015). In cod, temperature plays a significant role in the rate of morbidity and mortality of infections caused by the intracellular pathogen *Francisella noatunensis* subsp. *noatunensis*, where disease is observed only at temperatures above 14°C (Olsen et al., 2006; Mikalsen et al., 2009).

The presence and prevalence of bacterial infections in wild Atlantic cod are largely unexplored and knowledge of potential opportunistic pathogens is scarce. Experimental infection in Atlantic cod with the intracellular bacterium *Brucella pinnipedialis*, originally isolated from hooded seal (*Cystophora cristata*), did not cause disease in fish kept at 10°C. However, intracellular bacterial elimination was slow, suggesting that cod could be asymptomatic carriers of these bacteria in the marine environment (Nymo et al., 2016). Brucellosis is an important zoonotic disease in the terrestrial environment, and special attention is hence devoted to the pathological potential of the marine members of this genus. The epizootiology and pathogenicity of *Brucella* species isolated from seals are not fully understood, but transmission via the food web has been suggested (Lambourn et al., 2013; Nymo et al., 2013). Several seal species, including the hooded seal, prey upon the Atlantic cod (Bowen and Harrison, 1996; Mohn and Bowen, 1996; Haug et al., 2007).

Successful survival of a species in the marine environment is directly linked to its resistance against and tolerance to infections. Climate change driven migration of species will introduce new diseases through host or range shifts of known pathogens, while climate-mediated, physiological stresses may compromise host resistance and increase the frequency of opportunistic infections (Harvell et al., 1999). To explore how thermal stress can influence the immune response toward an opportunistic intracellular bacterial infection we housed Atlantic cod infected with *B. pinnipedialis* at normal seawater temperature (6°C) and at a temperature close to the thermal stress limit (15°C) for up to 7 weeks. We aimed to determine the effect of temperature on the rate of bacterial elimination, transcription of selected immune genes, production of specific antibodies, tissue infiltration of inflammatory cells, as well as the effect on general health status and survival. The influence of temperature on transmission of the infection was also evaluated by housing non-infected cohabitant cod with the respective infected groups.

## Materials and Methods

### Atlantic Cod (*Gadus morhua*)

**Figure 1:**
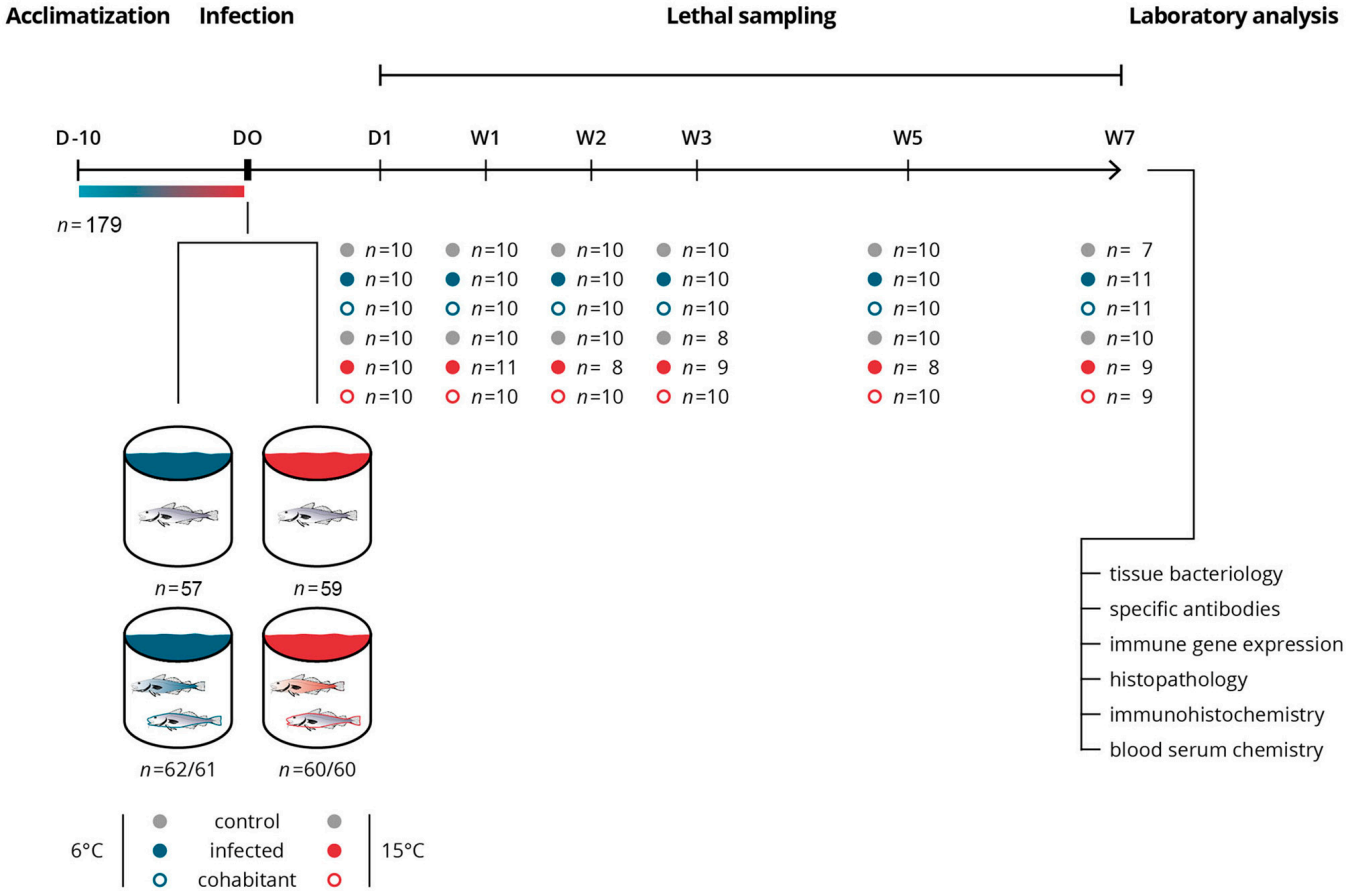
Schematic overview of the experimental infection study. The influence of water temperature on the immune response against an intracellular bacterial infection, *Brucella pinnipedialis*, was investigated in Atlantic cod (*Gadus morhua*). A total of 359 fish were included in the experimental challenge. Groups, treatments, sampling times, numbers of individual fish sampled, and ensuing laboratory analysis are shown in the illustration. Infected cod (filled blue and red circles) received an intraperitoneal injection of 1.25 × 10^8^ CFU of *B. pinnipedialis* hooded seal strain 17a-1. Control cod (gray circles) received sterile PBS. Cohabitant cod (open blue and red circles) were labeled on the right gill cover (operculum) and kept together with infected cod. A total of 8 fish died during the study, 5 in the infected group kept at 15°C, 1 in the cohabitant group kept at 15°C, 1 in the control group kept at 15°C, and 1 in the control group kept at 6°C.

Atlantic cod (*n* = 359, females and males) were purchased from the National breeding station for Atlantic cod (Tromsø, Norway) at approximately 100 g. The fish were kept at the Tromsø Aquaculture Research Station (Norway) in two 900 L tanks with filtered seawater and a 24 h light and feeding regime (Amber Neptun Starter 5.0 mm, Skretting, Stavanger, Norway) the first 10 days. During this period 50% of the fish were acclimatized from normal sea temperature (6°C) to 15°C. The fish were caught by a hand net, subsequently divided into experimental groups and kept in four 500 L tanks either at normal or elevated temperature with elsewise the same conditions (Figure 1). The fish were starved for 24 h and anesthetized with 0.08 g/L tricaine methane sulfonate (Western Chemical Inc., Ferndale, WA, USA) prior to infection and sampling. The protocol was approved by the Norwegian Animal Research Authority (permission no. 7265), and the experiments strictly followed the Norwegian Animal Welfare Act. All efforts were made to minimize suffering and stress during handling and sampling.

### Experimental Challenge

The experimental challenge included 6 groups (*n* ≈ 60 fish/group), namely control, infected, and cohabitant fish, kept at 6 and 15°C (Figure 1). In the infected groups (*n* = 122), each fish received 1.25 × 10^8^ *B. pinnipedialis* in 100 μl sterile phosphate-buffered saline (sPBS) intraperitoneally (ip). In the control groups (*n* = 116), 100 μl sPBS were injected ip. The cohabitant fish (*n* = 121) were labeled with visible implant elastomer (VIE) tags (Northwest Marine Technology, Shaw Island, WA, USA) injected subcutaneously on the right gill cover (operculum) and kept together with the infected fish. Fish (*n* = 7–10) from each of the experimental groups (control, infected, and cohabitant) were euthanized by an overdose of tricaine methane sulfonate at day 1 and week 1, 2, 3, 5, and 7 post infection (pi) for sampling. Power analysis (alpha = 0.05, power = 80%) was performed to determine the minimum sample size necessary to detect significant differences between groups. In line with ethical guidelines for animal experimentation, sample sizes were held at the lowest possible number to reduce the amount of animals used to answer the research question.

Some *Brucella* species are known to be highly pathogenic zoonotic bacteria and must be handled under biosafety level 3 conditions. The selected strain of *B. pinnipedialis* does not multiply in human macrophages or epithelial cells (Larsen et al., 2013b) and a reduced zoonotic capacity compared to other marine *Brucella* strains isolated from human cases (Brew et al., 1999; Sohn et al., 2003; McDonald et al., 2006) is suspected. Nevertheless, strict biosecurity routines were carefully followed during the entire experiment to avoid human infections and spillover to the environment.

### Bacteriology

In the experimental infection we used the *B. pinnipedialis* hooded seal (HS) strain 17a-1 (Tryland et al., 2005) which was stored at −80°C on Microbank™ beads (Pro-Lab Diagnostics, Round Rock, TX, USA) before use. To prepare the infectious doses one bead was plated and the bacteria were grown for 2–4 days and re-cultured for 96 h at 37°C. Bacteria were finally diluted in sPBS for the infective solution.

Samples of spleen, liver, heart, head kidney, back muscle, and blood were sterilely harvested from infected and cohabitant cod to quantify live bacteria. Euthanized cod were kept on ice until sampling was performed within 2 h. Dead fish were kept cold and sampled no longer than 4 h after identification. The samples were kept at −20°C until homogenized, serially diluted and plated to count the number of colony forming units (CFUs). Blood was collected from the caudal vein using vacutainer tubes without anticoagulant (BD Biosciences, San Jose, CA, USA) and plated (100 μl) directly on agar to evaluate the number of CFUs. Bacteria were grown on Tryptic Soy Agar (TSA) (Oxoid, Basingstoke, UK) at 37°C in air plus 5% CO_2_. Organ samples from dead fish were grown on TSA and modified Farrell [one vial of *Brucella* selective supplement (Oxoid) per liter of TSA + 5% fetal calf serum (FCS)] in parallel.

### Histology and Immunohistochemistry

Tissue samples of liver, spleen, and head kidney were fixed in 4% paraformaldehyde (PFA) in PBS, dehydrated, and embedded in paraffin. From each temperature, 3 control fish and the individuals with the highest (*n* = 2) and lowest (*n* = 2) CFUs in head kidney at each time point were selected for histological evaluation – in total 4 infected cod and 3 control fish were examined at day 1 and weeks 1, 2, and 3 (*n* = 56).

Paraffin sections (5 μm) were stained with hematoxylin and eosin (HE) or immune labeled for *Brucella* spp. Dewaxed, rehydrated sections were boiled in 10 mM sodium citrate, pH 6.0, in a microwave oven 2 × 5 min to demask antigens. After a blocking step using 2% TBSA (2% bovine serum albumin (BSA) in tris-buffered saline (TBS) with 0.05% Tween 20, pH 8.4), sections were incubated for 1 h at room temperature with a rabbit polyclonal anti-*Brucella* antibody (1:400, diluted in 2% TBSA, kindly provided by Prof. J.J. Letesson, Unité de Recherche en Biologie des Microorganismes, Laboratoire d'Immunologie et de Microbiologie, NARILIS, Université de Namur, Namur, Belgium) previously tested to be specific for *Brucella* spp., including *B. pinnipedialis* (Larsen et al., 2013b). Sections from non-infected control cods were used as negative control. Secondary antibody was Alexa Fluor 488 goat-anti-rabbit immunoglobulin (Ig)G (1:300, diluted in 2% TBSA, Molecular Probes, Life Technologies, Paisley, UK). Nuclei were visualized using 4′,6-diamidine-2′-phenylindole dihydrochloride (DAPI) (1:1,000 in PBS; Sigma-Aldrich, St. Louis, MO, USA). Evaluation of HE-sections was performed in a single-blind fashion where the lead pathologist was blinded. Fluorescence microscopy was performed using a Zeiss Axiophot photomicroscope equipped with incident-light fluorescence optics (Carl Zeiss, Oberkochen, Germany). Confocal microscopy was performed using a Zeiss LSM780 system equipped with a 63X NA1.4 oil immersion lens.

### Enzyme-Linked Immunosorbent Assay (ELISA)

Blood was collected from the caudal vein using vacutainer tubes without anticoagulant (BD Biosciences), and allowed to clot over night at 4°C. Serum was separated by centrifugation at 1,350 × *g* for 10 min and stored in cryogenic vials (Nalgene cryoware, Thermo Scientific, Rochester, NY, USA) at −20°C. The ELISA-test for anti-*Brucella* antibodies was performed as previously described (Nymo et al., 2016).

### Cytokine Gene Expression

Spleen samples were stored in RNAlater (Sigma-Aldrich, St. Louis, MO, USA) at −20°C. RNA was extracted by a Maxwell 16 using the Maxwell 16 LEV simplyRNA blood kit (Nerliens Meszansky, Oslo, Norway). The absence of genomic DNA was verified as previously described (Mikkelsen and Seppola, 2013). RNA quality and quantity were assessed by measuring absorbance at 230, 260, and 280 nm (Nanodrop 2000, Thermo Fisher Scientific Inc.). An A_260_/A_280_ ratio ≥2.0 and an A_260_/A_230_ ratio ≥2.1 were considered acceptable. The cDNA was made from 150 ng RNA (iScript™ cDNA Synthesis Kit, BioRad, Hercules, CA, USA). Primers for interleukin (IL)-1β, IL-10 (Seppola et al., 2008), interferon (IFN)-γ (Furnes et al., 2009), IL-12p40 (Mikkelsen et al., 2011), and ribosomal RNA (18S) (Seppola et al., 2008) were used as previously described. Quantitative reverse transcription PCR (RT-qPCR) was performed in duplicates of 8 μl cDNA diluted 1/30, 10 μl iTaq Universal SYBR Green Supermix (BioRad), 0.6 μl of each primer (10 μM), and 0.8 μl DEPC water (Invitrogen). Cycling parameters and calculation of threshold cycle (Ct) were as described (Seppola et al., 2007). Relative gene expression levels were quantified using the 2^−ΔΔ*CT*^ method (Livak and Schmittgen, 2001). Gene expression was calibrated against non-infected control cods kept at 6°C at the same time pi. The relative quantification values obtained from fish at the same time point were used to calculate the mean quantity ± standard error of the mean (SEM).

### Blood Serum Chemistry

Blood for clinical chemistry was collected in 2 ml tubes with clot activator (BD Biosciences) and sera were prepared as described for ELISA. Chemical analysis of serum samples was carried out at the Central Laboratory (Norwegian University of Life Sciences, Oslo, Norway) using an ADVIA 1800 Chemistry system (Siemens Healthcare GmbH, Erlangen, Germany). The clinical chemistry profile included aspartate aminotransferase (AST), alanine aminotransferase (ALT), alkaline phosphatase (AP), creatine kinase (CK), lactate dehydrogenase (LD), total protein, albumin, globulins, creatinine, cholesterol, triglycerides, free fatty acids, cortisol, calcium, chloride, potassium, and sodium concentrations. Reagents from Siemens Medical Solutions Diagnostics and Wako Chemicals (Neuss, Germany) were used for all analyses. Cortisol was measured using a solid-phase, competitive chemiluminescent enzyme immunoassay (Immulite® 2000 Cortisol) with an Immulite system (Siemens Healthcare GmbH). The method has been validated by the Central Laboratory for salmon, trout, halibut and cod. The validation study for cod comprised the analysis of 189 serum and heparinized plasma samples revealing an analytical range between < 28 nmol/L (the analytical detection limit) and > 1,380 nmol/L which is the upper calibration range. The analytical sensitivity was 5.5 nmol/L. The precision (CV) was below 10% both in the low and high analytical range.

### Statistical Analysis

The development of live body weights of cod over time was analyzed using general linear models. To ensure equality in variance the data was log-transformed. While significance values in the text refer to transformed data, estimates, and confidence intervals refer to back-transformed data for clarity. The overall survival was analyzed in a logistic regression approach by using general linear models with a binomial error structure. To reduce bias due to seven of eight deaths occurred among fish kept at 15°C, and hence stabilize estimates, we used a pseudo-data representation approach as described by Kosmidis and Firth (2009), library brglm in R, as well as grouping all combinations of treatments instead of interactions while analyzing (Gelman and Hill, 2007). We chose to group controls and cohabitants into one group to further reduce bias, as preliminary analyses showed no difference in survival rates (98.8, 95% confidence interval (CI) = [95.7, 100.0] vs. 98.8, 95% CI = [95.7, 100.0]). Time-specific survival over the experimental period was analyzed using a Kaplan-Meyer model (library survival in R) (Crawley, 2012). Statistical analyses of bacterial growth, gene expression, specific antibody response and blood serum chemistry were performed with a two-tailed, homoscedastic Student *t*-test (*p* < 0.05 was considered significant). When CFU counts in blood were compared to corresponding CFU counts in tissues a two-tailed, paired *t*-test was used.

## Results

### Viability of Fish Was Affected by the Combined Effect of Temperature and Infection

**Figure 2:**
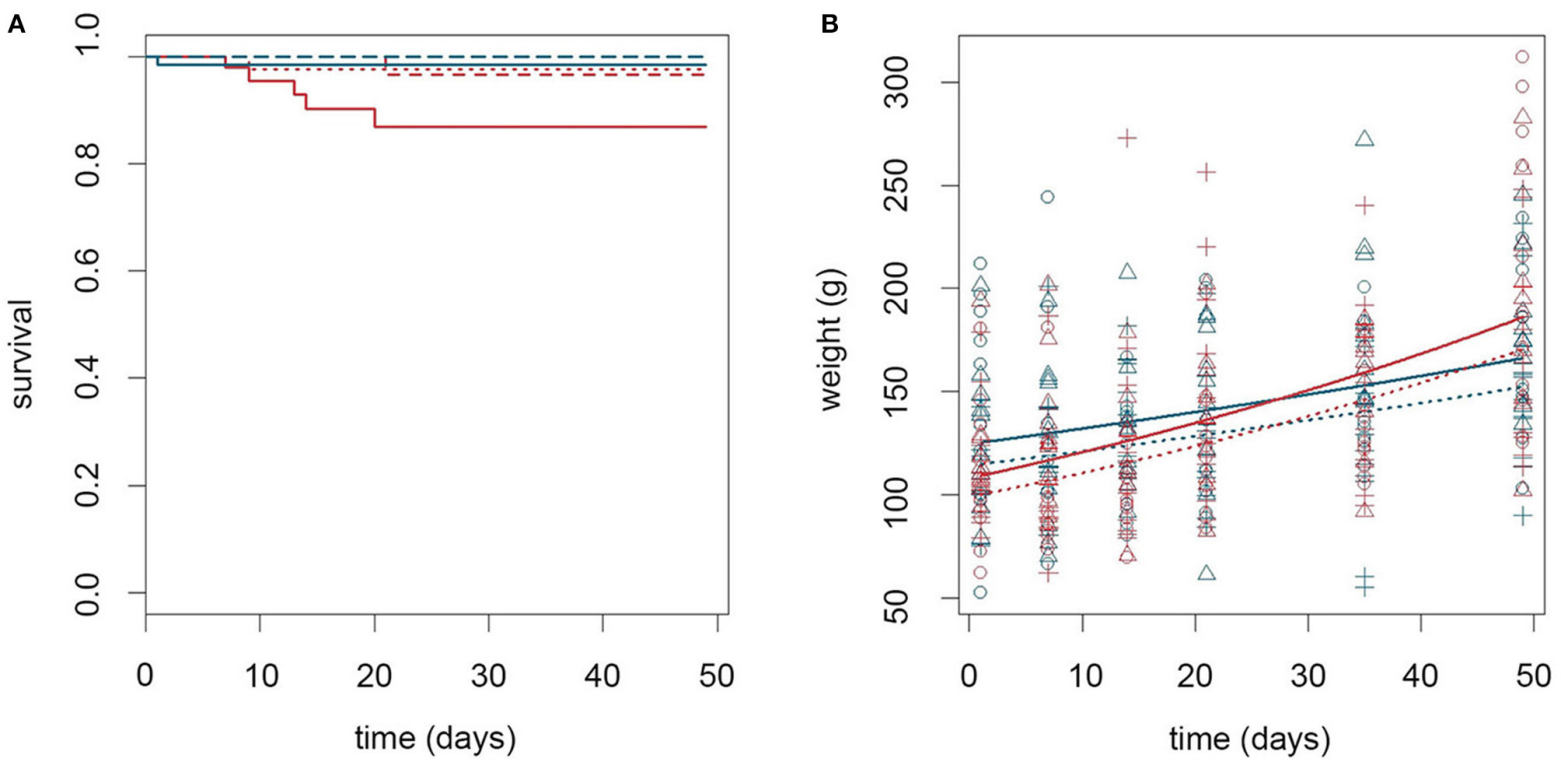
Temperature alone influenced growth rate while temperature and infection in combination affected viability. **(A)** Kaplan Meyer plot showing survival rates for cod during the experimental period. Line type indicate infection status (full = infected, dashed = non-infected cohabitants, and dotted = control). Color of lines indicate temperature (red = 15°C, blue = 6°C). **(B)** Scatter plot describing the growth of cod during the experimental period. Lines show estimated values, while symbols show observational data (o control, Δ infected, + cohabitant). Color and line type coding are similar to **(A)**. The dotted and dashed lines are completely overlapping in **(B)** and only the dotted line is shown to increase clarity.

The effect of thermal stress on the immune response toward an opportunistic intracellular bacterium, as well as growth and survival following bacterial challenge, was determined by housing infected fish at normal (6°C) and elevated (15°C) sea water temperature. Figure 2 shows the survival rates and growth curves during the experimental period. Five out of 60 infected fish kept at 15°C died between day 7 and 20 pi. This in contrast to one dead fish in each of the following groups: non-infected controls kept at 15°C (day 9), non-infected cohabitant cod kept at 15°C (day 21), and infected cod kept at 6°C (day 1) (Figure 2A). Infected fish kept at 15°C had an overall significant lower survival rate (91.0%, 95% CI = [82.1, 97.0]), than non-infected fish at both 6°C (99.6%, 95% CI = [97.3, 100.0], *p* = 0.034) and at 15°C (98.0%, 95% CI = [94.4, 99.7], *p* = 0.047), respectively. A significant effect of infection on viability was only observed in the high temperature group as infected fish kept at 6°C showed a lower, but not significant reduction in survival rate, compared to non-infected fish (97.6%, 95% CI = [91.8, 99.9], *p* = 0.283). No abnormal behavior or sign of disease were observed in the fish prior to death.

Analysis of body mass development revealed that infected cod weighed on average 11.9 and 12 grams more (*p* = 0.020 for both) than controls and non-infected cohabitants, respectively (calculated for day 24: 143.4 grams, 95% CI = [135.0, 152.4] vs. 131.5 grams, 95% CI = [123.7, 139.8], and 131.4 grams, 95% CI = [123.7, 139.6]). Temperature influenced the growth rate and both infected and non-infected cod grew faster at 15°C than at 6°C (interaction coefficient: 1.01 grams/day, 95% CI = [1.00, 1.01], *p* = 0.005, calculated for day 24) (Figure 2B).

### Clearance of *Brucella pinnipedialis* Was Most Effective at 15°C

**Figure 3:**
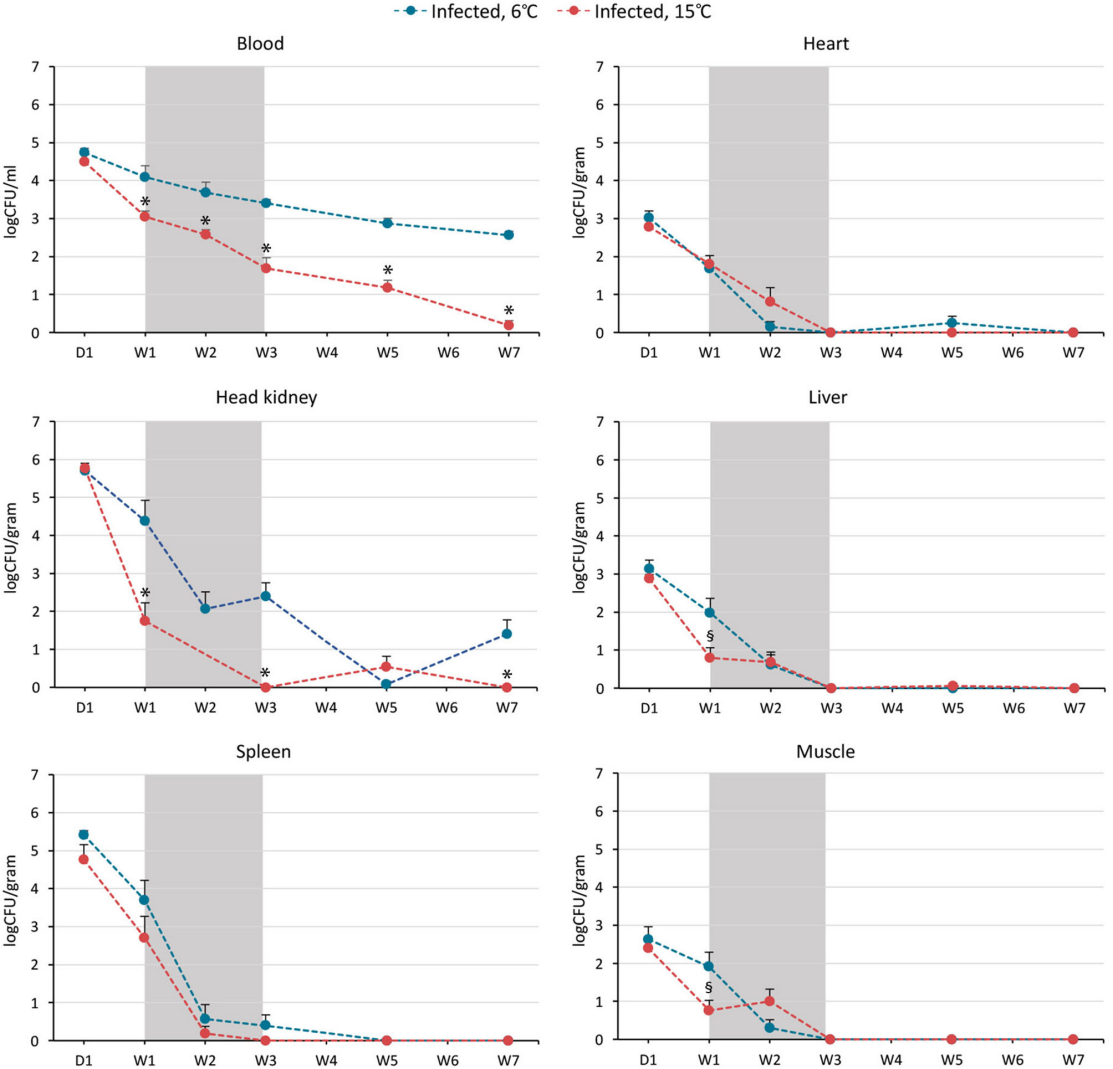
Elimination of the intracellular bacterium *Brucella pinnipedialis* was temperature dependent in Atlantic cod. Number of colony forming units (CFU)/ml blood and CFU/g heart, head kidney, liver, spleen, and muscle from Atlantic cod after intraperitoneal injection of 1.25 × 10^8^ CFU of *B. pinnipedialis* hooded seal strain 17a-1 per fish. Infected fish kept at 6°C (blue) and 15°C (red) were euthanized and sampled at day 1, week 1, 2, 3, 5, and 7 post infection. Each indicator shows the mean log CFU ± standard error of the mean of *n* = 7–11 fish (see Figure 1 and Supplementary Dataset 1). Gray shaded area represents the time span where mortality occurred in the 15°C infected group (survival data are presented in Figure 2). (*) CFU from infected cod kept at 15°C was significantly lower than CFU from infected cod kept at 6°C, *p* < 0.01. (§) CFU from infected cod kept at 15°C was significantly lower than CFU from infected cod kept at 6°C, *p* < 0.05.

Bacteriology showed that *B. pinnipedialis* caused a disseminated infection in the experimentally infected cod, whereas there was no bacterial growth from organs and blood from the cohabitant groups. On day 1 pi, viable bacteria were isolated from all organs investigated, including blood, from all infected fish, with no significant differences in the amount of CFUs retrieved in fish kept at 6 or 15°C (Figure 3). At week 1 pi, log CFUs (mean ± SEM) from head kidney, liver, muscle, and blood were significantly lower (*p* < 0.05) in infected cod kept at 15°C (1.75 ± 0.47, 0.80 ± 0.27, 0.76 ± 0.27, and 3.05 ± 0.15) compared to cod kept at 6°C (4.37 ± 0.55, 1.98 ± 0.39, 1.91 ± 0.38, and 4.10 ± 0.29). The number of CFUs retrieved from spleen was also lower in infected cod kept at 15°C, although not significant. Elimination of bacteria over time was observed in infected cod kept at both temperatures. By week 3, live bacteria were not isolated in head kidney, spleen, liver, heart and muscle of cod kept at 15°C, but were still cultured from head kidney and spleen of cod kept at 6°C. Bacteria could be isolated from blood at all times pi (day 1–week 7), but in significantly lower numbers in cod kept at 15°C for weeks 1, 2, 3, 5, and 7. Except for head kidney and spleen tissues on day 1 and at week 1, CFU counts in blood were significantly higher than in other tissues at corresponding time points and respective temperatures until week 7. At this time point, bacteria in the blood of cod kept at 15°C were almost completely eliminated with only two positive fish out of nine fish remaining (mean log CFUs 0.19 ± 0.13). In the 6°C infected group, viable bacteria were still isolated from all individuals at week 7 (mean log CFUs 2.56 ± 0.11).

Bacterial growth was not detected in spleen, head kidney, heart, liver or muscle of the five infected cod that died between week 1 and 3 in the warm-water group, suggesting rapid elimination of the infective dose in these individuals.

### Bacterial Degradation Was Detected Earlier in Atlantic Cod Kept at Higher Temperatures

**Figure 4:**
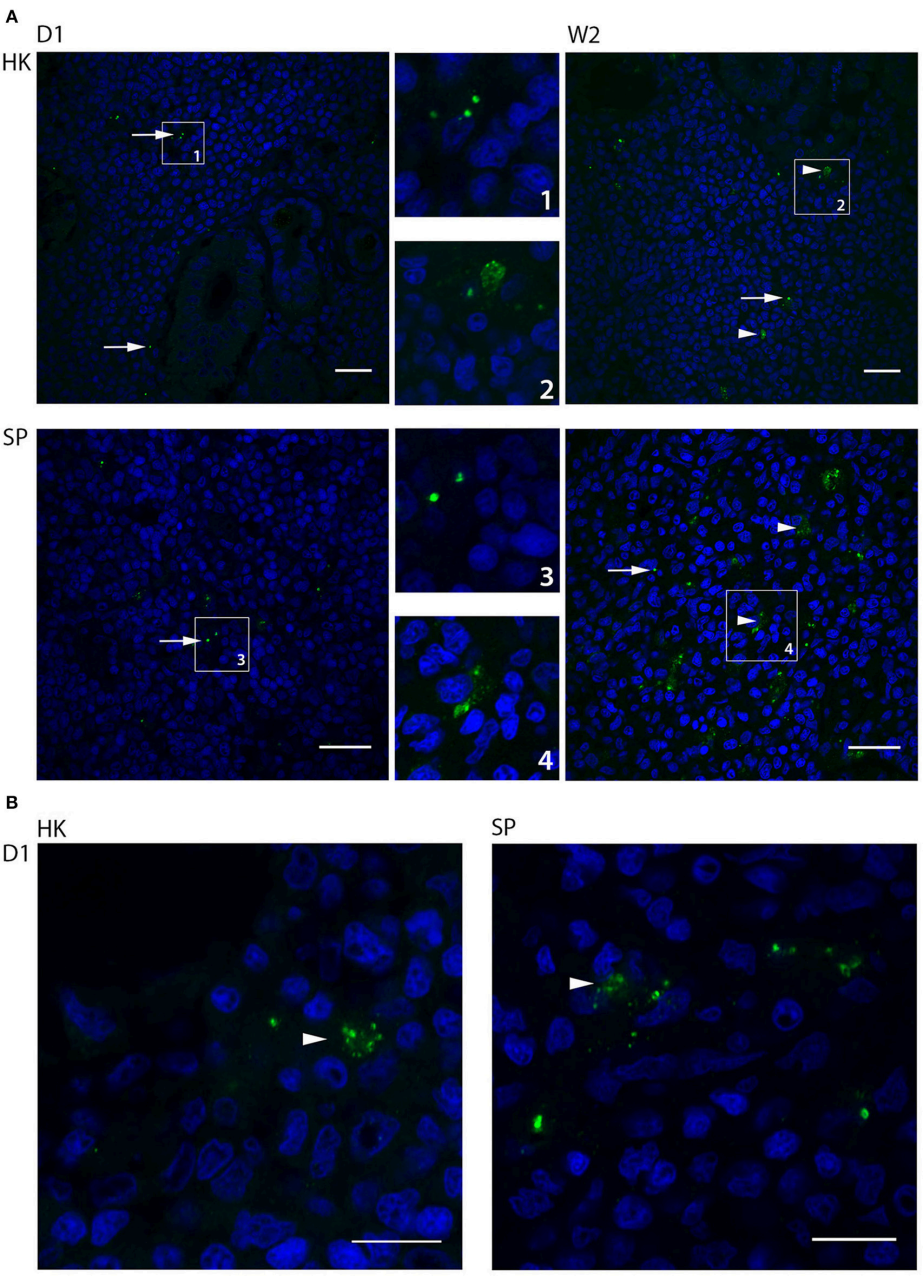
Intracellular localization and bacterial degradation of *Brucella pinnipedialis* in Atlantic cod head kidney and spleen. Paraffin sections of cod head kidney (HK) and spleen (SP) were labeled with a rabbit anti-*Brucella* antibody, and Alexa Fluor 488 goat-anti-rabbit IgG (green fluorescence). Cell nuclei were visualized with DAPI (blue). **(A)** Confocal microscopy of head kidney and spleen at day 1 (D1) and week 2 (W2) post infection in cod kept at 6°C. Intact bacteria (arrows) were observed inside cells both in head kidneys and spleen at day 1, while intact bacteria (arrows) and smaller particles (arrow heads), judged as bacterial debris, were detected in cells at week 2. Areas of interest are enlarged and numbered for identification. Scale bar 20 μm. Images shown in **(A)** are single images from associated Z-stacks shown in Supplementary information, Videos 1–4. **(B)** Early intracellular bacterial degradation in cod kept at 15°C. Confocal micrographs of head kidney and spleen show smaller particles (arrowheads), judged as bacterial debris, positively labeled with anti-*Brucella* antibody and Alexa Fluor 488-goat-anti-rabbit IgG (green fluorescence) already at day 1 post infection. Scale bar 10 μm.

Fluorescence- and confocal microscopy of immune labeled sections of head kidney, spleen and liver, using a polyclonal antibody toward *B. melitensis* that cross-react with *B. pinnipedialis* (Larsen et al., 2013a,b, 2016), showed uptake of *B. pinnipedialis* in scattered cells in all organs. Uptake was seen as immune stained granules inside the cells (Figure 4). The density of cells containing bacteria was most pronounced in head kidney and spleen, which are the macrophage-rich organs in fish, and immune labeled cells could be observed at all time points examined (day 1–week 3). In head kidney, cells with bacteria were often localized close to vascular structures, or sinusoids, in the hematopoietic interstitium between the nephrons, whereas in spleen, bacteria-containing cells were scattered throughout the pulp. The size of the immune labeled structures inside phagocytes varied from the expected size of *B. pinnipedialis* (Foster et al., 2007) to smaller particles suggestive of bacterial debris from intracellular degradation (Figure 4). Intracellular bacterial debris was observed already on day 1 in infected cod kept at 15°C (Figure 4B), whereas bacterial degradation was observed at later time points in cod kept at 6°C (Figure 4A). Immune labeling also revealed bacteria in head kidney and spleen from infected cod that were not culturable. This may represent bacteria destroyed or weakened by the cod's immune system. Loss of bacterial viability due to freezing and thawing prior to cultivation can also be a contributing factor, although not previously reported for other *Brucella* strains.

Evaluation of HE-stained sections of head kidney, spleen, and liver from infected cod (day 1 and weeks 1, 2, and 3 pi) and controls showed no clear difference between the groups, suggesting that the infection with *B. pinnipedialis* did not cause specific pathology.

### Expression of Immune Genes in Atlantic Cod Was Correlated With Water Temperature and Infection With *Brucella pinnipedialis*, Both Alone and in Combination

**Figure 5:**
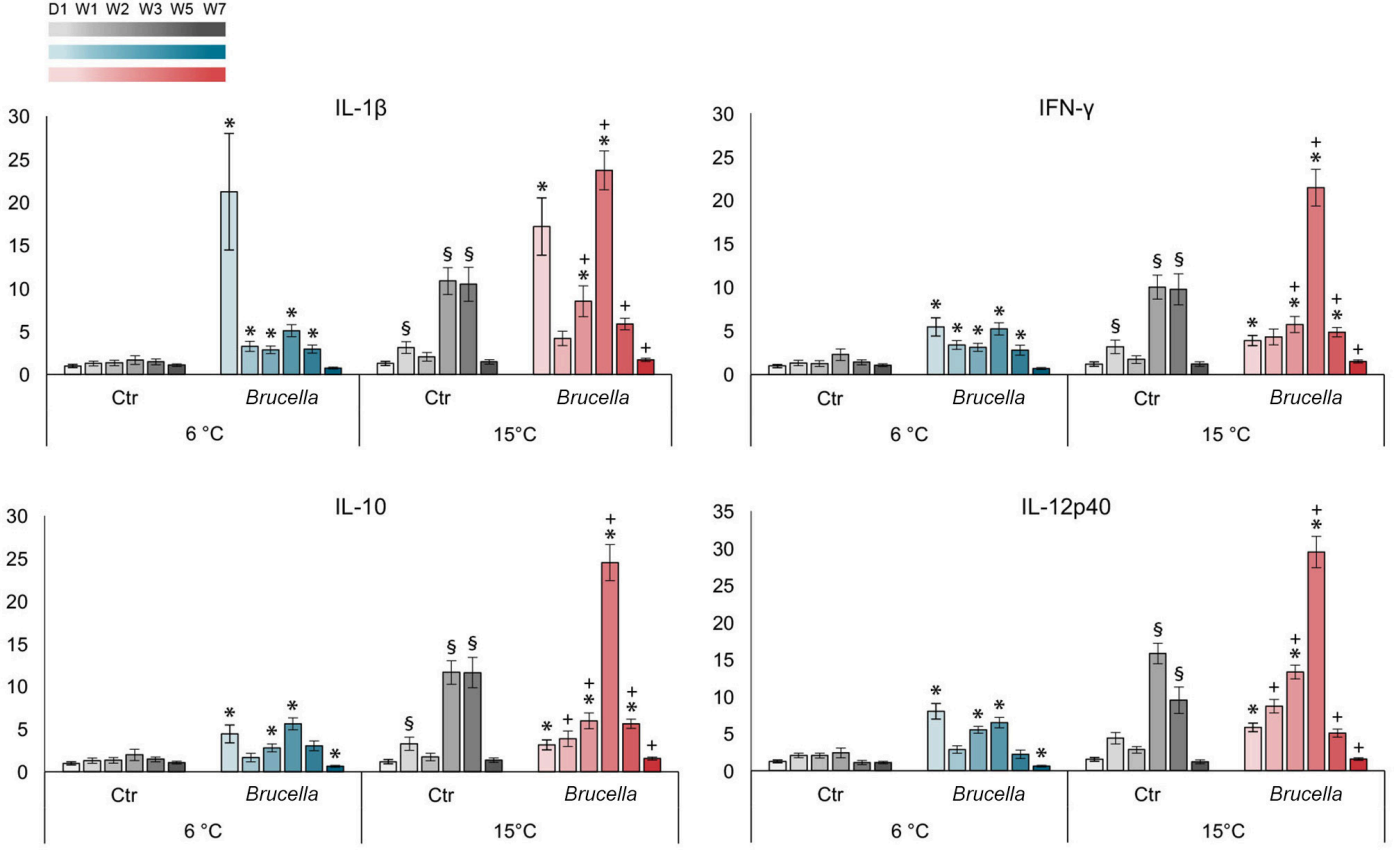
Expression of selected immune genes was influenced by both temperature and bacterial infection. Relative gene expression in fold changes of interleukin (IL)-1β, interferon (IFN)-γ, IL-10, and IL-12p40 in spleen from infected cod kept at 6°C and 15°C on day 1 and week 1, 2, 3, 5, and 7 post infection. The gene expression was normalized against the housekeeping gene 18S ribosomal RNA and calibrated against saline injected control cods kept at 6°C. Bars show the mean ± standard error of the mean of *n* = 7–10 fish (see Figure 1 and Supplementary Dataset 2). (*) Significantly different from control cod kept at corresponding temperature. (§) Significantly different from control cod kept at 6°C. (+) Significantly different from infected cod kept at 6°C, *p* < 0.05.

Expression of selected immune genes, IL-1β, IFN-γ, IL-10, and IL-12p40, was measured in spleen from control and infected cod kept at 6 and 15°C using RT-qPCR (Figure 5). Results showed that gene expression was positively correlated with water temperature, and significantly correlated with infection, adjusted for water temperature.

Increased water temperature alone yielded escalated expression of the immune genes investigated, with significant 3-fold increases at week 1 and 10-fold increases at weeks 3 and 5 in the levels of IL-1β, IFN-γ, and IL-10, and a 15-fold and a 9-fold rise at weeks 3 and 5 of IL-12p40, in control cod kept at 15°C compared to control cod at 6°C (Figure 5).

Significantly increased expression of all genes was also observed for infected cod kept at both temperatures when compared with their corresponding non-infected control groups, but the pattern of increment was different (Figure 5). Infected fish kept at 6°C showed an immediate response with high levels of immune gene transcription on day 1, most pronounced was a 21-fold rise in IL-1β. Subsequently, there was a reduction in gene transcription to nearly background levels at week 1, followed by a 5-fold rise in all gene transcripts at week 3. From this point in time the gene expression decreased and a significant down-regulation was observed for IL-10 and IL-12p40 at week 7. The initial increase in gene expression on day 1 was more moderate for infected cod kept at 15°C with a 17-fold rise in IL-1β as the maximum (Figure 5). From week 2 onwards, all investigated genes were significantly higher expressed in infected cod kept at 15°C compared to infected cod kept at 6°C. For infected cod kept at 15°C, the most pronounced rise in gene expression was detected at week 3 with peaks of up to 30-folds that were in strong contrast to both non-infected cod kept at 15°C and infected cod kept at 6°C. A subsequent decline toward week 7 was observed for all genes investigated; however, no down-regulation was observed in this group.

### The Specific Antibody Response Toward *Brucella pinnipedialis* Was Temperature-Dependent

**Figure 6:**
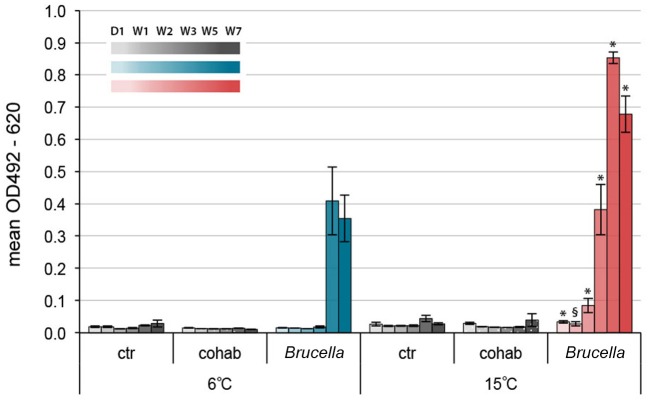
Specific antibody response toward *Brucella pinnipedialis* in Atlantic cod was temperature dependent. Levels of anti-*Brucella* antibodies in Atlantic cod kept at 6°C (blues) and 15°C (reds) on day 1 and week 1, 2, 3, 5, and 7 after intraperitoneal injection of 1.25 × 10^8^ CFU of *B. pinnipedialis* hooded seal strain 17a-1. Control cod (grays) received sterile PBS. Cohabitant cod (dotted grays) were labeled on the right operculum and kept together with infected cod. Each bar shows the mean optical density ± standard error of the mean of *n* = 7–11 fish (see Figure 1). (*) Significantly different from infected cod kept at 6°C, *p* < 0.01. (§) Significantly different from infected cod kept at 6°C, *p* < 0.05.

Figure 6 shows the ELISA-test results for *Brucella* spp. antibodies in serum samples. In the 6°C group, antibody end-point titers for *Brucella* spp. given as optical density (OD)_492−620_ (mean ± SEM), were significantly increased in infected cod compared to control cod kept at the same water temperature at week 5 and 7 (Figure 6 and Supplementary Table 1). Mean OD_492−620_ values of infected cod kept at 15°C increased earlier and were significantly higher than those of control cod kept at the same water temperature already in week 2. Interestingly, OD_492−620_ values of infected cod kept at 15°C were significantly higher than for infected cod kept at 6°C for all time points pi. There were no significant increases in OD_492−620_ values for cohabitant cod compared to control cod at the respective temperatures.

### Organ Physiology Was Influenced by Infection and Increased Water Temperature

Levels of cellular enzymes (AST, ALT, AP, CK, LD), metabolites (total protein, albumin, globulin, creatinine, triglycerides, free fatty acids), electrolytes (Ca^2+^, Na^+^, K^+^, Cl^−^), and hormones (cortisol) in serum of infected and control cod were determined (Table 1). Normal values for these serum components in juvenile Atlantic cod are not validated. Hence, all parameters herein are compared to the respective control groups, and in between infected groups, without establishing what may be assumed as the normal range according to age and season of the year. The levels of nearly all parameters were higher on day 1 pi compared to week 2 at the respective temperature (Table 1), both for control and infected fish. This general effect may have been caused by the sedation and/or stress applied on day 0 in relation to inoculation and labeling. There were significant differences in serum parameters between control groups at 6°C and 15°C, both on day 1 and at week 2 (Table 1 and Supplementary Table 2). Results further showed a significant effect of infection, adjusted for water temperature (Table 1), indicating that homeostasis was also affected by the *B. pinnipedialis* infection. In addition, significant differences were identified between infected cod at 6 and 15°C which demonstrate that the effect of infective stress on homeostasis differs according to water temperature. Most variations in the measured parameters were modest and mean values of the infected groups were often within the range of values presented in the corresponding control groups (Table 1). However, for some parameters, and most pronounced two weeks pi, the mean values of the infected groups were outside the corresponding control range (calcium and chloride at 6°C and AST, ALT, and sodium at 15°C). Significant increases in AST and ALT at week 2 pi indicates liver and/or muscle cell damage. Infection and elevated water temperature in combination, as well as elevated water temperature alone, but not infection alone, influenced potassium homeostasis. Cortisol levels in control cod were significantly higher at 6°C on day 1 and at week 2 pi compared to at 15°C. Also, infected cod at 6°C had significantly higher serum cortisol levels than infected cod at 15°C on day 1 pi. Mean cortisol levels were increased in infected cod compared to respective control cod at both time points and temperatures, although not significant, suggesting that cortisol was more influenced by water temperature than infection.

**Table 1:** Influence of water temperature and infection on selected physiological parameters.

		**Day 1**				**Week 2**			
		**Control**		**Infected**		**Control**		**Infected**	
**Parameter**	**Unit**	**Mean**	**Range**	**Mean**	**Range**	**Mean**	**Range**	**Mean**	**Range**
**6**°**C**									
AST	U/L	7.6	5–11	7.7	5–11	4.8	3–8	^A^ 6.6	4–10
ALT	U/L	5.2	3–10	5.5	0–9	3.7	3–6	3.8	0–7
AP	U/L	58.1	29–89	59.0	40–78	30.8	18–54	36.1	22–53
CK	U/L	2.3	0–19	0.6	0–6	0.0	0–0	0.0	0–0
LD	U/L	379.4	162–765	434.1	182–658	55.7	12–133	94.7	47–213
Total protein	g/L	31.4	25–35	33.1	27–39	27.6	25–31	28.5	24–32
Albumin	g/L	15.5	13–18	16.1	13–21	13.0	11–15	13.8	12–16
Globulin	g/L	15.9	11–19	17.0	14–21	14.6	13–17	14.7	12–17
A/G ratio		1.0	0.8–1.3	1.0	0.8–1.2	0.9	0.8–1.2	0.9	0.9–1.1
Creatinine	μmol/L	102.5	45–228	149.2	51–241	79.2	46–135	^A^ 125.2	95–180
Cholesterol	mmol/L	7.5	5.8–8.9	7.7	6.7–9.0	6.7	6.0–7.2	^A^ 7.2	6.3–8.2
Triglycerides	mmol/L	7.2	5.5–8.6	6.5	5.2–7.2	5.7	4.8–6.9	6.2	5.1–7.5
Free fatty acids	mmol/L	6.6	6.4–7.3	6.8	6.4–7.2	6.9	6.6–7.0	6.8	6.6–7.1
Ca	mmol/L	2.5	2.0–2.9	2.3	1.6–2.7	2.1	1.4–2.5	^A^ 2.6	2.3–2.8
Na	mmol/L	153.7	144–161	158.3	142–171	163.1	158–167	161.4	158–165
K	mmol/L	21.1	15.0–26.8	23.2	13.7–32.6	12.1	7.7–16.2	13.5	11.0–17.4
Na/K ratio		7.7	5.4–10.6	7.3	4.4–12.0	14.5	9.8–21.7	12.1	9.1–15.0
Cl	mmol/L	99.7	93–114	^A^ 128.3	117–148	98.9	93–106	^A^ 111.5	107–116
Cortisol	nmol/L	1108.6	753–1380	1276.7	1082 – 1380	624.9	298 – 1068	752.3	157–1380
**15**°**C**									
AST	U/L	9.4	7–14	^A^ 7.6	6–10	6.0	4–8	^AC^ 8.6	6–11
ALT	U/L	5.2	2–10	5.9	4–10	^B^ 2.2	1–5	^A^ 5.8	3–9
AP	U/L	43.1	26–65	55.4	35–94	32.1	21–41	36.4	14–62
CK	U/L	2.6	0–19	0.0	0–0	0.5	0–5	0.9	0–7
LD	U/L	^B^ 163.7	35–454	^C^ 89.1	36–176	52.3	24–101	65.1	4–142
Total protein	g/L	30.6	23–41	^C^ 28.7	25–31	28.1	25–33	28.0	13–38
Albumin	g/L	14.9	10–23	^C^ 13.9	12–16	^B^ 11.7	10–14	12.9	5–16
Globulin	g/L	15.7	11–20	^C^ 14.8	12–18	^B^ 16.4	14–19	15.1	8–24
A/G ratio		1.0	0.7–1.3	0.9	0.7–1.1	^B^ 0.7	0.6–0.8	0.9	0.6–1.3
Creatinine	μmol/L	^B^ 186.0	100–256	^A^ 127.5	94–167	70.0	17–173	^A^ 130.0	50–239
Cholesterol	mmol/L	6.4	3.6–7.7	7.1	6.0–8.2	6.4	5.1–8.1	6.3	2.4–8.7
Triglycerides	mmol/L	^B^ 4.7	1.1–6.2	5.7	4.4–7.9	5.1	4.0–7.5	^C^ 4.3	1.9–5.9
Free fatty acids	mmol/L	6.8	3.4–7.4	6.9	6.6–7.2	^B^ 7.1	6.7–7.4	7.0	5.9–7.4
Ca	mmol/L	2.5	2.1–3.1	2.5	1.7–2.9	2.3	1.1–2.8	2.3	1.2–2.9
Na	mmol/L	156.8	148–167	^A^ 161.7	155–166	164.9	160–169	171.3	163–212
K	mmol/L	^B^ 16.4	11.1–23.7	^AC^ 12.4	8.8–14.7	^B^ 5.6	1.4–13.6	^C^ 6.5	3.8–8.9
Na/K ratio		^B^ 9.9	6.2–15.0	^AC^ 13.6	8.7–18.3	^B^ 41.7	11.8–119.3	^C^ 28.3	19.8–43.9
Cl	mmol/L	^B^ 127.5	120–140	127.0	115–148	^B^ 119.2	117–122	121.9	113–164
Cortisol	nmol/L	^B^ 593.2	173–814	^C^ 845.9	248–1297	^B^ 357.4	130–858	682.7	249–1380

*Blood serum chemistry of control and infected cod at day 1 and week 2 post infection. Results are presented as mean of n = 8–10 cod in each group (see Figure 1). Range is also given, as normal values for healthy juvenile Atlantic cod are not published. ^A^Significantly different from non-infected control cod kept at the same temperature. ^B^Significantly different from non-infected control cod kept at 6°C. ^C^Significantly different from infected cod kept at 6°C, p < 0.05. Significance levels are shown in Supplementary Table 1*.

## Discussion

In the present study we show that the cod immune response against an opportunistic pathogen, *B. pinnipedialis*, were strongly influenced by the environmental temperature. In cod kept at temperatures close to their thermal tolerance limit, the overall responses to the infection was of higher amplitude and the reduction of viable bacteria was more pronounced in most organs investigated, particularly in blood. Interestingly, despite superior clearance rates, a significant number of infected cod died within a restricted period of time in the warm water group. This observation is intriguing, as the Atlantic cod, along with many other marine organisms, will have to endure warmer water temperatures according to future climate scenarios. Exposure to novel opportunistic and pathogenic microorganisms is very likely to occur and the future health status of aquatic organisms will be challenged. Higher prevalence rates and increased pathogenicity of fish pathogens due to variation in water temperature is well known in fish (Green and Haukenes, 2015). However, it was not known how temperature would affect the outcome of opportunistic infections in cod. The results presented herein highlight that it is essential to gain a deeper insight into how the immune system adapts to increased temperatures and how the total energy cost of an effective immune response affect the overall health of the cod.

Host defense against pathogens has two forms: resistance and tolerance (Soares et al., 2017). Resistance is the ability of the host to limit a pathogen burden, whereas tolerance is the ability to limit the negative consequences of infection. Host survival during infection requires a delicate balance between host defense, which is essential for the detection and elimination of pathogens and host tolerance, which is critical for minimizing collateral tissue damage and maintenance of internal homeostasis. When evaluating the observed immune responses, our results will in the following sections be discussed in relation to resistance and tolerance.

In mammals, thermal regulation of immunity through the cardinal symptom fever confers a survival benefit in the course of infection (Evans et al., 2015). Febrile temperatures boost the effectiveness of the immune response by stimulating both innate and adaptive immunity, hence increasing host resistance. Although not capable of increasing their body temperature above the surrounding water temperature, teleost fish display behavioral fever by seeking warmer environments during infection (Rakus et al., 2017). Simulated behavioral fever enhanced the expression of the pro-inflammatory cytokine IL-1β in rainbow trout (Gräns et al., 2012). We also observed a temperature effect on the gene expression of IL-1β in Atlantic cod; both alone and in combination with infection, with the most pronounced effect between weeks 2-5 pi. IFN-γ, IL-10, and IL12p40 followed a similar pattern with increased gene expression in both control and infected cod kept at 15°C. The augmented levels of pro-inflammatory cytokines following increased water temperature may contribute to a stronger immune response toward *B. pinnipedialis*. In contrast to previous short-term studies suggesting that increased water temperature has a limited effect on the Atlantic cod antibacterial response (Hori et al., 2013), we show that the temperature-induced increased transcription of selected cytokines also persisted over time. The results from both temperature groups further corroborates that cytokine gene expression following challenge with the opportunistic *B. pinnipedialis* is induced to a similar extent as seen during obligate pathogen exposure (Ellingsen et al., 2011), even in the absence of specific disease symptoms.

Tissue damage control is an important part of disease tolerance and programmed cell death is triggered if earlier damage control mechanisms fail to preserve the function of parenchymal cells (Soares et al., 2017). Macrophage production of cytokines that limit the extent of inflammatory reactions, like IL-10, is associated with clearance of damaged and dying cells. Compared to infected cod kept at 6°C, expression of IL-10 between weeks 1–7 was significantly higher in infected cod kept at 15°C, indicating increased activation of anti-inflammatory mechanisms to reduce potential tissue damage following immune activation.

As part of the first line of defense against invading pathogens (Secombes, 1996), the response of phagocytic cells to increased temperatures may have an impact on disease resistance. Our bacteriological investigations showed that the number of bacteria was greater in organs known to be rich in phagocytic cells, such as head kidney and spleen, and immunohistochemistry revealed that bacteria were located in cells in areas known to be occupied by macrophages. From our previous work we know that *B. pinnipedialis* can survive in cod head kidney monocytes/macrophages for at least 48 h without major reduction in viable bacteria (Nymo et al., 2016). Our results showed that elimination of *B. pinnipedialis* was fastest in cod kept at 15°C. A contributing factor to this observation is likely increased bactericidal activity in phagocytic cells. Respiratory burst activity of cod phagocytes was shown to increase at both 10 and 15°C compared to 4°C (Nikoskelainen et al., 2006). Furthermore, cod lysosomal α-mannosidase is temperature sensitive and showed highest enzymatic activity at 37°C (Sørensen et al., 2001), suggesting more effective intralysosomal degradation of endocytosed/ phagocytosed ligands in cod at higher temperatures. This is supported by the notion that intracellular degradation of endocytosed procollagen type I propeptide in cod scavenger endothelial cells was more effective at 12°C than at 4°C (Sørensen et al., 1998). This leads us to hypothesize that intralysosomal enzymatic activity, and hence lysosomal degradation of ligands, is more efficient in cod kept at temperatures around 12°C, and possibly at 15°C, than in cod kept at 6°C, despite the former temperature being close to the upper thermal limit.

However, *B. pinnipedialis* infected fish in the high temperature group showed early signs of tissue damage, as evidenced by a significant increase in serum AST and ALT levels at week 2 pi compared to infected fish kept at 6°C, and non-infected controls. This suggests that the anti-oxidant systems were insufficient. Host-induced damage by oxidative stress caused by the production of reactive oxygen species (ROS), e.g., during innate immune responses, requires tolerance mechanisms and a sufficient amount of antioxidant scavenging compounds must be available upon activation of the immune system to prevent damage to the host (Ayres and Schneider, 2012).

Because of the important role of immunoglobulins (Ig) in the protection against infections, the effect of environmental temperature on Ig production has been investigated in several fish species, including Atlantic cod. The concentration and activity of natural antibodies (IgM) in serum of non-stimulated cod increased when water temperature was raised from 1 to 14°C (Magnadóttir et al., 1999; Bowden, 2008). Our results were in coherence with these observations and showed that the production of specific antibodies against *B. pinnipedialis* was also influenced by temperature, with earlier onset and stronger intensity of the response in infected cod kept at 15°C.

Activation of the immune system is energy demanding and although efficient for combating infections, the induction of fever occurs at a high metabolic cost (Lochmiller and Deerenberg, 2000). The amount of energy consumed during immune activation has not yet been determined in cod, but it is well known that resistance to, recovery from, and coping with stressors can be energy intensive for fish (Schreck and Tort, 2016). For comparison, a rise of 1°C in body temperature in endothermic animals requires a 10–12.5% increase in metabolic rate (Evans et al., 2015). A protracted immune response will consume more energy. It may also lead to excessive tissue damage initiating energy demanding cellular repair processes. As important as mounting an immune response toward foreign organisms is the subsequent control and down-regulation of cascading inflammatory events and return to homeostasis. Production of a larger immune response than necessary is perhaps the most obvious sort of tolerance failure in infections (Ayres and Schneider, 2012). Our immune gene expression analysis showed that the transcription levels of selected genes were elevated for a longer period of time in cod kept at 15°C, suggesting a larger energy consumption related to transcription and translation to proteins. Interestingly, the peak of gene expression between weeks 2 and 3 pi correlated well with the deaths observed in the infected, high temperature group. Due to rapid tissue decomposition, samples for evaluation of immune gene expression were not collected from the deceased fish. However, based on the fact that no live *B. pinnipedialis* were isolated from any organs investigated, it is tempting to speculate that immune activation was overwhelming leading to subsequent effective bacterial elimination in these individuals, but also to fatal outcomes.

When evaluating host protection against infection it is often difficult to disentangle disease tolerance from resistance to infection. However, an overall assessment of host homeostasis and direct quantification of parameters that estimate tissue function plotted against host pathogen load may reveal variations in disease tolerance (Soares et al., 2017). At similar, or even lower, bacterial load, cod kept at 15°C had inferior health judged by the detected electrolyte imbalance, the increased serum levels of liver enzymes, as well as the observed deaths. These observations suggest that a reduced disease tolerance occurred in the warm water group.

In addition to modulating the immune response, the environmental temperature will also affect other physiological processes in fish. Transcriptome analysis of liver in rainbow trout subjected to heat stress revealed that several pathways, including those for protein metabolism and energy metabolism, as well as immunity, were influenced (Li et al., 2017). As pointed out by Hanna et al. (2008) “*environmental conditions can exert profound effects on the physiology of an organism and often manifest as changes in the metabolism*.” A 2.7-fold increase in both standard (resting fish) and active (fish forced to swim) metabolic rate was shown for Atlantic cod at 10°C compared to those at 2°C (Claireaux et al., 2000), while another study found a 2.2-fold increase in standard metabolic rate, but only a 1.4-fold increase in active metabolic rate for Atlantic cod evaluated at 15°C compared to those at 5°C (Schurmann and Steffensen, 1997). Metabolic rate was not investigated in the present study, but serum chemistry analysis showed that temperature affected the homeostasis of different electrolytes, in particular potassium. Both control and infected cod kept at 15°C revealed significantly lower values of potassium in the second week pi compared to cod kept at 6°C. It is worth noting that the serum samples were stored over night at 4°C which might have contributed to increased levels of potassium (Mayer and Donnelly, 2013), leading to an overestimation and the possibility of the real values being even lower. The reported values should therefore solely be compared to the control samples in our study, which were treated similarly. Hypokalemia may be due to loss of potassium through the kidneys in mammals, but infectious diseases are also supposed to be a major cause in fish (Mayer and Donnelly, 2013). Temperature, but not infection, was correlated with hypokalemia in our material. Electrolyte imbalance can have detrimental effects on the cardiovascular system with hypertension and ventricular arrhythmias as the most severe aftermath (Weiner and Wingo, 1997).

Cortisol has several established physiological roles in osmoregulation, metabolism, growth, stress, and immune function in teleosts (McCormick, 2011). Previous research in juvenile Atlantic cod has shown acute thermal stress to be a stronger driver for increased cortisol compared to long-term increases in water temperature (Pérez-Casanova et al., 2008a,b). Contrary to what may be expected, we observed that infected and non-infected cod kept at 15°C displayed significantly lower levels of cortisol compared to cod kept at 6°C. Hypokalemia is shown to reduce the conversion of corticosterone to aldosterone in rats (Baumann and Müller, 1972). The mineral corticoid aldosterone is the primary regulator of salt and water balance in mammals. Fish lack CYP11B2 aldosterone synthase and cannot produce aldosterone (Kiilerich and Prunet, 2011). In the absence of aldosterone, cortisol has an important osmoregulatory function in fish. It is not known whether changes in the serum potassium concentration can affect cortisol levels, but it is tempting to hypothesize that these two factors are interdependent. However, our study does not support any conclusions and the connection between osmoregulation, low potassium levels and cortisol in cod needs to be further investigated.

For an organism with indeterminate growth, like cod, 41% of the life-time energy is spent on growth (West et al., 2001). Growth is also affected by temperature. Growth rates in juvenile Atlantic cod kept at 7 and 14°C were significantly higher than in cod kept at 1°C, and sexual maturity occurred earlier at higher temperatures (Magnadóttir et al., 1999). During our 7 weeks long experimental infection we observed that juvenile cod kept at 15°C grew faster compared to cod kept at 6°C. None of the cod reached sexual maturity during the study period, but in the natural environment this would be a factor to consider when evaluating multiple synchronous energy demanding processes.

In their natural environment, animals usually have limited access to nutritional resources. Constraints created by the amount of energy allocated to fuel the immune response may create trade-offs between immune function and other fitness-related traits like growth, reproduction and thermoregulation (Lochmiller and Deerenberg, 2000). Resistance responses can be expensive in terms of energy expenditure and profound changes in energy distribution occur upon infection. Disruption of these changes can also reduce tolerance if not enough energy is left for repair processes (Schneider and Ayres, 2008). Infection did not have any significant impact on growth rate in our study, indicating that the increased growth observed in cod kept at the highest temperature was not impeded by the activated immune response. This suggests a lack of trade-off between growth and immune responses in our material, and one may speculate if this have contributed to the significantly increased death rate observed in infected cod kept at 15°C.

The results presented herein highlight the importance of timely balanced immune responses. Our study is the first to describe death following concomitant temperature stress and infection despite efficient clearance of an opportunistic bacterial infection. In addition to diseases caused by pathogenic microbes, health and well-being of fish may also be hampered by non-pathogenic microbes. We show here that immune activation at increased water temperature, close to the thermal limit of the species, resulted in an efficient immune response toward an opportunistic pathogen; however, the combined amount of energy spent on resistance and tolerance, growth and physiological homeostasis may have contributed to increased mortality.

The North Sea cod stock has been in poor condition for the last decades, experiencing drastic population declines most probably due to overfishing (Sobel, 1996; ICES, 2006; Engelhard et al., 2014). Although registered improvements, nine smaller coastal stocks are still listed as vulnerable (MCS, 2015). Sustainable management of Atlantic cod in the future requires our awareness of potentially additive, synergistic or antagonistic health effects induced by environmental stress. Unsuccessful adaptation to chronic stressors can result in allostatic overload and mortality (Sneddon et al., 2016). In light of current and predicted climate change, it is of utmost importance to increase our knowledge on how temperature stress affects the energetic cost of immune activation in ectotherms and how disease resistance and tolerance are influenced by unfavorable physical environmental conditions.

## Data Availability

The authors declare that all the data supporting the findings of this study are available within the article and its Supplementary Information files and from the corresponding author upon reasonable request.

## Author Contributions

AL, IN, SA, MJ, and JG conceived and designed the experiments. AL, IN, MJ, and JG performed the experimental infection. AL, IN, MJ, and KS performed laboratory analyses. AL, IN, MS, RR, and KS analyzed the data. AL wrote the paper. KS and RR wrote sections of the manuscript. All authors provided critical evaluation of the manuscript, read and approved the submitted version.

### Conflict of Interest Statement

The authors declare that the research was conducted in the absence of any commercial or financial relationships that could be construed as a potential conflict of interest. The reviewer TE declared a past co-authorship with the authors MJ and SA to the handling editor.
