# Supplementary material for: Concomitant Temperature Stress and Immune Activation may Increase Mortality Despite Efficient Clearance of an Intracellular Bacterial Infection in Atlantic Cod
Source: Front Microbiol. 2018 Dec 4;9:2963. doi: 10.3389/fmicb.2018.02963 (PMC6289035; doi:10.3389/fmicb.2018.02963)
Supplement: Supplementary file 1 [file Table_1.DOCX]

***Supplementary material***

**Concomitant Temperature Stress and Immune Activation may Increase Mortality Despite Efficient Clearance of an Intracellular Bacterial Infection in Atlantic Cod.**

Anett K. Larsen ^1, *^, Ingebjørg H. Nymo ^1,§^, Karen K. Sørensen ^2^, Marit Seppola ^2^, Rolf Rødven ^3^, María Pilar Jiménez de Bagüés ^4^, Sascha Al Dahouk ^5^, Jacques Godfroid ^1^

^1^ Arctic Infection Biology, Department of Arctic and Marine Biology, UiT – The Arctic University of Norway, Tromsø, Norway.

^2^ Department of Medical Biology, UiT – The Arctic University of Norway, Tromsø, Norway

^3^ Department of Research and Development, UiT – The Arctic University of Norway, Tromsø, Norway.

^4^ Unidad de Tecnología en Producción y Sanidad Animal, Centro de Investigación y Tecnología Agroalimentaria, Instituto Agroalimentario de Aragón (CITA-Universidad de Zaragoza), Zaragoza, Spain

^5^ German Federal Institute for Risk Assessment, Berlin, Germany

^§^ Current address: Norwegian Veterinary Institute, Tromsø, Norway

* Correspondence: Anett K. Larsen, [anett.k.larsen@uit.no](mailto:anett.k.larsen@uit.no)

**Supplementary Video 1. Intracellular localization of *Brucella pinnipedialis* in head kidney.** Confocal micrographs of head kidney (HK) at day 1 showed intact bacteria labeled with anti-*Brucella* antibody and Alexa Fluor 488 goat-anti-rabbit IgG (green fluorescence). Cells were labeled with DAPI (blue) for visualization of nuclei. The video is a compilation of Z-stack images taken with a step size of 0.5 μm and shows bacteria scattered throughout the tissue. The picture shown in Figure 4B is picture number 4, of in total 9, in the Z-stack.

**Supplementary Video 2. Intracellular localization of *Brucella pinnipedialis* in head kidney.** Confocal micrographs of head kidney (HK) at week 2 showed intact bacteria and smaller particles, judged as bacterial debris, labeled with anti-*Brucella* antibody and Alexa Fluor 488 goat-anti-rabbit IgG (green fluorescence). Cells were labeled with DAPI (blue) for visualization of nuclei. The video is a compilation of Z-stack images taken with a step size of 0.5 μm and shows bacteria scattered throughout the tissue. The picture shown in Figure 4B is picture number 4, of in total 9, in the Z-stack.

**Supplementary Video 3. Intracellular localization of *Brucella pinnipedialis* in spleen.** Confocal micrographs of spleen at day 1 showed intact bacteria labeled with anti-*Brucella* antibody and Alexa Fluor 488 goat-anti-rabbit IgG (green fluorescence). Cells were labeled with DAPI (blue) for visualization of nuclei. The video is a compilation of Z-stack images taken with a step size of 0.5 μm and shows bacteria scattered throughout the tissue. The picture shown in Figure 4B is picture number 1, of in total 9, in the Z-stack.

**Supplementary Video 4. Intracellular localization of *Brucella pinnipedialis* in spleen.** Confocal micrographs of spleen at week 2 showed intact bacteria and smaller particles, judged as bacterial debris, labeled with anti-*Brucella* antibody and Alexa Fluor 488 goat-anti-rabbit IgG (green fluorescence). Cells were labeled with DAPI (blue) for visualization of nuclei. The video is a compilation of Z-stack images taken with a step size of 0.5 μm and shows bacteria scattered throughout the tissue. The picture shown in Figure 4B is picture number 5, of in total 9, in the Z-stack.

| Time  post infection | **OD _492-620_** | | | | | |
| --- | --- | --- | --- | --- | --- | --- |
|  | 6 °C | | | 15 °C | | |
|  | control | cohabitant | infected | control | cohabitant | infected |
| D1 | 0.02 ± 0.00 | 0.02 ± 0.00 | 0.02 ± 0.00 | 0.03 ± 0.01 | 0.03 ± 0.00 | 0.03 ± 0.00 |
| W1 | 0.02 ± 0.00 | 0.01 ± 0.00 | 0.02 ± 0.00 | 0.02 ± 0.00 | 0.02 ± 0.00 | 0.03 ± 0.01 |
| W2 | 0.01 ± 0.00 | 0.01 ± 0.00 | 0.01 ± 0.00 | 0.02 ± 0.00 | 0.02 ± 0.00 | 0.09 ± 0.02 |
| W3 | 0.01 ± 0.00 | 0.01 ± 0.00 | 0.02 ± 0.00 | 0.02 ± 0.00 | 0.02 ± 0.00 | 0.38 ± 0.08 |
| W5 | 0.02 ± 0.00 | 0.01 ± 0.00 | 0.41 ± 0.11 | 0.04 ± 0.01 | 0.02 ± 0.00 | 0.85 ± 0.02 |
| W7 | 0.03 ± 0.01 | 0.01 ± 0.00 | 0.36 ± 0.07 | 0.03 ± 0.00 | 0.04 ± 0.02 | 0.68 ± 0.06 |

**Supplementary Table 1.** **Mean OD 492-620 ± standard error of the mean (SEM) in the ELISA-test.** OD_620_ for all samples analyzed was 0.04 ± 0.00 indicating limited interference from non-specific components. The OD_620_ of the sample diluting buffer was 0.04 ± 0.00 and the OD_492_ was 0.05 ± 0.00 demonstrating a low background in the ELISA assay applied.

| Parameter | ***p*-value** | | | | | | | |
| --- | --- | --- | --- | --- | --- | --- | --- | --- |
|  | Day 1 | | | | Week 2 | | | |
|  | **inf vs.**  **ctr 6°C** | **inf vs.**  **ctr 15°C** | **inf 6°C vs. 15°C** | **ctr 6°C**  **vs. 15°C** | **inf vs.**  **ctr 6°C** | **inf vs.**  **ctr 15°C** | **inf 6°C**  **vs. 15°C** | **ctr 6°C**  **vs. 15°C** |
| **AST** | 0.915 | **0.033** | 0.895 | 0.074 | **0.024** | **0.002** | **0.031** | 0.058 |
| **ALT** | 0.803 | 0.512 | 0.730 | 1.000 | 0.912 | **3E-04** | 0.101 | **0.012** |
| **AP** | 0.911 | 0.104 | 0.548 | 0.071 | 0.302 | 0.474 | 0.995 | 0.764 |
| **CK** | 0.400 | 0.199 | 0.305 | 0.913 | 1.000 | 0.701 | 0.304 | 0.331 |
| **LD** | 0.522 | 0.137 | **3E-06** | **0.013** | 0.078 | 0.483 | 0.449 | 0.812 |
| **Total protein** | 0.337 | 0.355 | **0.002** | 0.717 | 0.361 | 0.968 | 0.800 | 0.629 |
| **Albumin** | 0.503 | 0.439 | **0.011** | 0.636 | 0.136 | 0.348 | 0.431 | **0.032** |
| **Globulin** | 0.351 | 0.404 | **0.010** | 0.877 | 0.880 | 0.434 | 0.838 | **0.016** |
| **A/G ratio** | 0.519 | 0.912 | 0.890 | 0.596 | 0.309 | 0.068 | 0.413 | **5E-04** |
| **Creatinine** | 0.119 | **0.005** | 0.305 | **0.002** | **0.001** | **0.041** | 0.623 | 0.620 |
| **Cholesterol** | 0.527 | 0.148 | 0.086 | 0.056 | **0.027** | 0.871 | 0.144 | 0.384 |
| **Triglycerides** | 0.108 | 0.083 | 0.059 | **2E-04** | 0.163 | 0.216 | **0.004** | 0.248 |
| **Free fatty acids** | 0.192 | 0.742 | 0.217 | 0.674 | 0.775 | 0.398 | 0.399 | **0.023** |
| **Ca** | 0.183 | 0.744 | 0.141 | 0.944 | **0.003** | 0.918 | 0.120 | 0.501 |
| **Na** | 0.195 | **0.018** | 0.357 | 0.240 | 0.189 | 0.245 | 0.097 | 0.185 |
| **K** | 0.379 | **0.009** | **1E-04** | **0.019** | 0.216 | 0.511 | **1E-06** | **3E-04** |
| **Na/K ratio** | 0.690 | **0.006** | **1E-04** | **0.036** | 0.111 | 0.250 | **3E-05** | **0.013** |
| **Cl** | **4E-06** | 0.893 | 0.858 | **3E-09** | **5E-07** | 0.628 | 0.079 | **7E-11** |
| **Cortisol** | 0.052 | 0.092 | **0.003** | **1E-04** | 0.425 | 0.103 | 0.763 | **0.031** |

**Supplementary Table 2. Significant differences between groups in blood serum chemistry.** Differences between groups (*n* = 8-10 fish/group, see figure 1) were examined by a two-tailed, homoscedastic Student *t*-test. Infected cod were compared to control cod at the respective temperature, infected cod at 6°C were compared to infected cod at 15°C, and control cod at 6°C were compared to control cod at 15°C. *P*-values < 0.05 are considered significant and indicated by bold numbers.

**Supplementary Dataset 1. Cultivable bacteria in selected tissues of Atlantic cod after *in vivo* infection with *Brucella pinnipedialis* at 6 and 15°C.** The number of cultivable bacteria in blood, heart, head kidney (HK), spleen, liver, and muscle of infected cod were determined at day 1 and weeks 1, 2, 3, 5, and 7 post infection. The excel sheet lists CFU/ml blood and CFU/gram tissue for heart, HK, spleen, liver, and muscle. Information about sample times post infection (column A), water temperature (column B), experimental groups (column C), and fish id (column E) are also given. Individuals excluded from the assay are listed in column S and the reason for exclusion is described in column T.

**Supplementary Dataset 2. Expression of immune genes in Atlantic cod after *in vivo* infection with *Brucella pinnipedialis* at 6 and 15°C.** RT-qPCR was performed to investigate the expression of selected immune genes (interleukin (IL)-1β, interferon (IFN)-γ, IL-10, and IL-12p40) in spleen of saline injected control cod and cod infected with *B. pinnipedialis*. Ct values obtained at day 1 and weeks 1, 2, 3, 5, and 7 post infection are listed in the excel sheet. Information about sample times post infection (column A), water temperature (column B), experimental groups (column C), and fish id (column E) are also given. The positive control was created by pooling cDNA extracted from 10 infected fish in the warm water group (spleen, day 1 post infection) and included on all plates to evaluate interplate variability. No-template control (NTC) was constituted of all reaction components except cDNA, which was replaced with nuclease-free (DEPC) water. Individuals excluded from the assay are listed in column Q and the reason for exclusion is described in column R.
